# Characterization of *FLT3*-ITD^mut^ acute myeloid leukemia: molecular profiling of leukemic precursor cells

**DOI:** 10.1038/s41408-020-00352-9

**Published:** 2020-08-25

**Authors:** Serena Travaglini, Daniela Francesca Angelini, Valentina Alfonso, Gisella Guerrera, Serena Lavorgna, Mariadomenica Divona, Anna Maria Nardozza, Maria Irno Consalvo, Emiliano Fabiani, Marco De Bardi, Benedetta Neri, Fabio Forghieri, Francesco Marchesi, Giovangiacinto Paterno, Raffaella Cerretti, Eva Barragan, Valentina Fiori, Sabrina Dominici, Maria Ilaria Del Principe, Adriano Venditti, Luca Battistini, William Arcese, Francesco Lo-Coco, Maria Teresa Voso, Tiziana Ottone

**Affiliations:** 1 Department of Biomedicine and Prevention, University of Tor Vergata, Rome, Italy; 2 Santa Lucia Foundation, I.R.C.C.S., Neuro-Oncohematology, Rome, Italy; 3 Ematologia, Ospedale S. Eugenio, Dipartimento di Biomedicina e Prevenzione, Rome, Italy; 4 University of Modena and Reggio Emilia, Azienda Ospedaliera di Modena, Modena, Italy; 5 Hematology and Stem Cell Transplant Unit, IRCCS Regina Elena National Cancer Institute, Rome, Italy; 6 Hospital Universitari i Politècnic La Fe, Valencia, Centro de Investigación Biomédica en Red de Cáncer, Madrid, Spain; 7 Diatheva srl, via Sant’Anna 131, 61030 Cartoceto, (PU) Italy

**Keywords:** Haematopoietic stem cells, Translational research

## Abstract

Acute myeloid leukemia (AML) with *FLT3*-ITD mutations (*FLT3*-ITD^mut^) remains a therapeutic challenge, with a still high relapse rate, despite targeted treatment with tyrosine kinase inhibitors. In this disease, the CD34/CD123/CD25/CD99+ leukemic precursor cells (LPCs) phenotype predicts for *FLT3*-ITD-positivity. The aim of this study was to characterize the distribution of *FLT3*-ITD mutation in different progenitor cell subsets to shed light on the subclonal architecture of *FLT3*-ITD^mut^ AML. Using high-speed cell sorting, we sequentially purified LPCs and CD34+ progenitors in samples from patients with *FLT3*-ITD^mut^ AML (*n* = 12). A higher *FLT3*-ITD^mut^ load was observed within CD34/CD123/CD25/CD99+ LPCs, as compared to CD34+ progenitors (CD123+/−,CD25−,CD99low/−) (*p* = 0.0005) and mononuclear cells (MNCs) (*p* < 0.0001). This was associated with significantly increased CD99 mean fluorescence intensity in LPCs. Significantly higher *FLT3*-ITD^mut^ burden was also observed in LPCs of AML patients with a small *FLT3*-ITD^mut^ clones at diagnosis. On the contrary, the mutation burden of other myeloid genes was similar in MNCs, highly purified LPCs and/or CD34+ progenitors. Treatment with an anti-CD99 mAb was cytotoxic on LPCs in two patients, whereas there was no effect on CD34+ cells from healthy donors. Our study shows that *FLT3-*ITD mutations occur early in LPCs, which represent the leukemic reservoir. CD99 may represent a new therapeutic target in *FLT3*-ITD^mut^ AML.

## Introduction

Acute myeloid leukemia (AML) is characterized by heterogeneous genetic abnormalities, which significantly influence clinical outcome. Therefore, the definition of molecular profiles is essential at the time of initial diagnosis, to identify specific biomarkers and to design specific treatments^1^. Although available treatments induce complete remission (CR) in about 80% of AML patients^2^, 30–40% of them will eventually relapse, due to the emergence of resistant clones.

Multiple genetic abnormalities have shown to account for the clinical heterogeneity of AML and, according to the WHO 2016 classification and the 2017 revised European Leukemia Net (ELN) guidelines^3^, in addition to karyotype assessment, mutational analyses are required for proper AML classification. Internal tandem duplications of the fms-like tyrosine kinase 3 (*FLT3)* gene (*FLT3*-ITD^mut^) characterize ~25% of AML and represent an independent predictor of poor prognosis, being associated with an increased risk of relapse^3,4^. The *FLT3*-ITD^mut^ allelic ratio (AR), reflecting the *FLT3*-ITD^mut^ leukemic burden, also plays a significant role, especially in the context of *NPM1* mutations, where AR < 0.5 or ≥0.5, define favorable or intermediate prognostic categories^3^.

*FLT3*-ITD is an unstable marker during AML evolution, with *FLT3*-ITD^mut^ patients at diagnosis becoming negative or acquiring the mutation at relapse in 25% and 10% of cases, respectively^5,6^. These data indicate that *FLT3*-ITD^mut^ testing must be confirmed at the time of disease recurrence to eventually add *FLT3*-targeted drugs^3,7^.

We identified minor *FLT3*-ITD^mut^ subclones at the time of initial AML diagnosis, expressing the CD34/CD123/CD25/CD99+ immunophenotype, in 83% of cases with a *FLT3*-ITD^mut^ relapse^8,9^. These leukemic precursor cells (LPC) *FLT3*-ITD^mut^ subclones may expand at disease recurrence, and their identification and characterization represent an important challenge. Indeed, *FLT3*-ITD mutations have been shown to occur in primitive AML cells^10^, representing early drivers of leukemogenesis mainly in patients with a normal karyotype, while additional cooperating mutations may occur later, contributing to disease progression^11,12^.

In this line, to shed light on the subclonal architecture of *FLT3*-ITD^mut^ AML and to track *FLT3*-ITD^mut^ and other clones, we characterized the molecular profile of CD34/CD123/CD25/CD99+ enriched LPCs.

## Patients and methods

### Patient characteristics

We studied 38 patients with a *FLT3*-ITD^mut^ AML, aged 21–82 years, diagnosed at the Hematology Unit of Policlinico Tor Vergata in Rome, during the years 2016–2019. AML patients aged <75 years received intensive chemotherapy according to the European Organization of Research and Treatment of Cancer (EORTC)/Gruppo Italiano Malattie EMatologiche dell’Adulto (GIMEMA) protocols, while patients aged >75 years received supportive care or hypomethylating treatment. Treatment included the addition of tyrosine kinase inhibitors (TKIs) in four patients (three received Midostaurin and one Gilterinib), relapsed at a median of 10.5 months from initial diagnosis (range 5–14 months). According to the declaration of Helsinki, all patients gave informed consent for the study, which was approved by the Institutional Review Board of the Policlinico Tor Vergata.

### Genetic characterization

Morphologic, immunophenotypic, and molecular routine analysis were carried out on all 38 AML cases at diagnosis and eventually at relapse. For genetic analysis, total RNA was extracted from Ficoll-Hypaque isolated bone marrow (BM) mononuclear cells (MNCs), which contained over 80% of the blast population, and reverse-transcribed using standard procedures^13^. DNA was extracted from BM-MNCs collected at diagnosis, and for selected cases during follow-up, using a column-based protocol (Qiagen, Hilden, Germany).

Mutations of *NPM1* and *FLT3* genes were investigated at the DNA and RNA level on MNCs isolated from AML samples and on purified cell populations^8,14^. As reported elsewhere^3^, the cut-off for *FLT3*-ITD^mut^ positivity was AR ≥ 0.05. The validity of this method for quantification of the mutation burden was confirmed by the linear relationship between the value of the AR to the proportion of mutated versus total *FLT3* (ITD + WT) allele (*n* = 99 samples, *R* = 0.6, *p* < 0.0001, data not shown). *NPM1* molecular analysis was carried out on all AML samples included in this study^15^ and the *NPM1* mutation load was calculated using the same criteria adopted for *FLT3*-ITD^mut^ AR determination. Amplification products for *FLT3*-ITD^mut^ and *NPM1* genes were analysed on the Applied Biosystems 3130 Genetic Analyzer (Thermo Fisher Scientific), using the GeneMapper™ Software 5.

Patients’ mutational profile was studied in 12 *FLT3*-ITD^mut^ AML cases at diagnosis, on DNA extracted from BM-MNCs, using NGS and the targeted Oncomine Myeloid-Research Assay (Thermo Fisher Scientific) (Table 1). The assay includes the entire coding region of 17 genes (*ASXL1, BCOR, CALR, CEBPA, ETV6, EZH2, IKZF1, NF1, PHF6, PRPF8, RB1, RUNX1, SH2B3, STAG2, TET2, TP53, ZRSR2*, and the “hotspot” region of 23 genes *(ABL1, BRAF, CBL, CSF3R, DNMT3A, FLT3, GATA2, HRAS, IDH1, IDH2, JAK2, KIT, KRAS, MPL, MYD88, NPM1, NRAS, PTPN11, SETBP1, SF3B1, SRSF2, U2AF1*, and *WT1)*. Panel yields 526 amplicons with an average length of 400 bp. Following the manufacturer’s instructions and run in the Ion 530™ chip on the Ion Torrent S5 instrument (Thermo Fisher Scientific), sequence analysis were performed using the Ion Torrent Suite Software v.5.8.0 and the Ion Reporter software v.5.10.3.0 and v.5.10.5.0. Analysis of the raw sequencing data and alignment of sequencing reads to the reference genome (Human genome build 19 of Hg 19) was performed by the Ion Reporter software for Ion S5. Identification of sequence variants was carried out by Ion Torrent Variant Caller Plugin software v4.4-r76860, and coverage of each amplicon was determined by the Coverage Analysis Plugin software v4.4-r77897. The minimum coverage depth was set at 400X for somatic DNA variants. The sensitivity of this NGS assay was 5% variant allele fraction (VAF). 5′ and 3′ untranslated regions (UTRs), intronic donor splice-site variants, and polymorphisms were filtered out.

**Table 1:** Clinical and biological features of AML patients at diagnosis.

UPN	Sex	Age	Karyotype	*FLT3-*ITD^mut^ allele ratio (AR) in MNCs	Other genetic alterations	BM-blasts (%)	WBC (10^9^/L)	Relapse (months)
1	M	63	46,XY	0.03	–	67	25.4	8
2	M	40	46,XY	0.06	*NPM1*^mut^ (type A) *IDH2* (R140Q)	25	13.5	5
3	F	58	n.v	0.6	*IDH2* (R140Q)	80	51.7	/
4	M	79	46,XY	0.27	–	42	35.0	5
5	F	66	46,XX	0.1	–	72	12.0	3
6	F	67	46,XX	0.36	*NPM1*^mut^ (type A)	83	35.6	Death in induction
7	F	64	46,XX	0.04	–	81	58.6	/
8	F	69	46,XX,inv(16)(p13q22)	0.05	*CBFB/MYH11*	90	7.8	/
9	F	57	46,XX, +8	0.03	*IDH2* (R140Q)	80	6.6	5
10	M	68	46,XY	0.38	*NPM1*^mut^ (type A)	20	20.3	7
11	M	58	46,XY	0.6	–	87	46.4	Death in induction
12	F	59	nv	0.55	–	69	32.1	Death in induction

*MNCs* mononuclear cells.

To assess the mutational profile of AML patients who relapsed after TKIs treatment, we compared the genetic asset of four patients at diagnosis and at relapse (Table 2), studying 30 genes known to be frequently mutated in myeloid malignancies, using NGS and the Myeloid Solution by SOPHiA GENETICS (Saint-Sulpice, Switzerland). The resulting captured libraries were further processed on a MiniSeq sequencing platform (Illumina, San Diego, CA, USA). FASTQ sequencing files were then uploaded on the SOPHiA DDM platform for analysis by the specific technology. Only mutations identified as highly or potentially pathogenic were considered for analysis. The sensitivity of this NGS method was ≥1%. Further details on the NGS analysis have been previously reported^16^.

**Table 2:** Molecular features of AML patients relapsed after tyrosine kinase inhibitors (TKIs) treatment.

	BM blasts (%)	*NPM1*	*FLT3*-ITD^mut^	*FLT3*-ITD^mut^ AR	Size *FLT3*-ITD^mut^ clone	Relapse (months)	TKI treatment
UPN13							
Diagnosis	20	Wild-type	mut	0.05	31 bp	5	Midostaurin
Relapse	70	Wild-type	Wild-type	/	/		
UPN14							
Diagnosis	83	mut	mut	0.75	17 bp; 81 bp	13	Midostaurin
Relapse	74	mut	mut	3.97	81 bp		
UPN15							
Diagnosis	70	mut	mut	0.44	40 bp; 60 bp	14	Midostaurin
Relapse	30	mut	mut	0.2	40 bp		
UPN16							
Diagnosis	62	mut	mut	0.4	57 bp	8	Quizartinib
Relapse	85	mut	mut	0.42	57 bp		

### Multiparametric flow cytometry (MPFC) studies and high-speed cell sorting

Immunophenotypic studies were performed at diagnosis using standard techniques, as detailed elsewhere^17^. After completion of diagnostic tests, further sorting, and molecular analysis were performed in particular on 12 AML patients with available cells at diagnosis. The percentage of CD123/CD25/CD99+ cells within the CD34+ cell fraction was studied as previously reported by our group^9^. Immunophenotype studies were carried out using a CytoFLEX flow cytometer (Beckman Coulter), and data were analysed using the FlowJo software (TreeStar Inc. Ashland, USA). MNCs were isolated by Ficoll Hypaque and were stained with the following anti-human antibodies: CD45 APC-vio770, CD99R PE (purchased from Miltenyi Biotec), CD33 Fitc (e-Bioscience), CD117 PE-Cy7, CD14 PE-Cy5.5 (purchased from Beckman Coulter), CD38 PE-CF594, CD3 Brilliant Violet 785, CD25 Brilliant Violet 421 and CD123 Brilliant Violet 605 (purchased from Becton Dickinson). Progenitor cell populations were sorted by high-speed cell sorting, in the 6-way sorting MoFlo Astrios (Beckman Coulter), using a sequential gating strategy to separate CD34/CD123/CD25/CD99+ LPCs, CD38+ and CD38− cells, CD34+ progenitors (CD123+/−,CD25−,CD99 low/−) and T-lymphocytes (CD45high/CD3+/CD14−/CD33−/CD34). For analysis of CD99 analysis, measurements included the evaluation of the number of cells positively stained and the mean fluorescence intensity (MFI) on leukemic blasts (adjusted for background fluorescence using negative internal controls).

### In vitro anti-CD99 mAb treatment assays

To determine whether CD99 may be a relevant therapeutic target, we investigated the ability of a monoclonal antibody (mAb) against CD99 to induce direct cytotoxicity on enriched LPCs. The anti-CD99 mAb clone 3.2–3 (Diatheva, Cartoceto, PU, Italy) is a fully human recombinant IgG4 monoclonal antibody, specific for the extracellular domain of CD99. CD34/CD123/CD99+/CD25− enriched-LPCs sorted from 2 AML patients who relapsed after Midostaurin/chemotherapy treatment and CD34+ cells from 2 healthy donors were seeded at 1 × 10^5^ cells/well in 200 µl of Stemspan SFEM II medium supplemented with CD34+ Expansion Supplement (10×) (Stem Cell Technologies, Carlsbad, California), in a flat-bottom 96-well plate. The anti-CD99 mAb diluted in sterile PBS was then added at a concentration of 25 μg/ml, and cells were incubated for 72 h at 37 °C, in 5% CO_2_. At the end of the incubation time, cells were stained with a LIVE/DEAD fixable dead cell stain (Life Technologies,Carlsbad,California), according to the manufacturer’s instructions, and analyzed by flow cytometry.

### Statistical analysis

Statistical analysis was performed using the GraphPad Prism version 5.0 (La Jolla, CA, USA). *P* values < 0.05 were considered statistically significant. Differences between categorical variables were evaluated by the Pearson’s chi-squared test, while differences between continuous variables were analysed by unpaired *t*–test with 95% confidence intervals.

## Results

### Heterogeneity of FLT3-ITD^mut^ AML at diagnosis and relapse

**Fig. 1:**
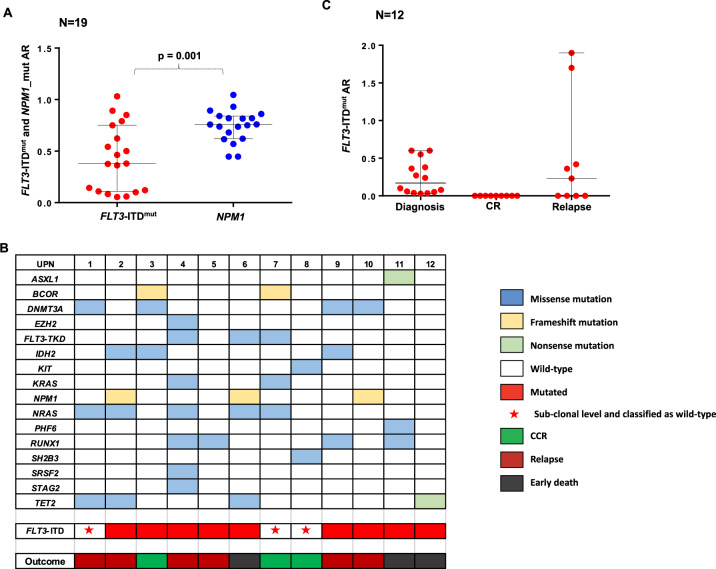
Molecular characterization of *FLT3*-ITD^mut^ AML patients. **a** *FLT3*-ITD^mut^ and *NPM1*^mut^ AR analysis on MNCs in double-mutant AML patients at diagnosis (*n* = 19). The *FLT3*-ITD^mut^ and *NPM1*^mut^ AR were defined as the ratio of the area under the curve of *FLT3*-ITD^mut^ and *FLT3* wild-type alleles. **b** Targeted NGS analyses performed in primary blasts from *FLT3*-ITD^mut^ AML cases at diagnosis (*n* = 12). **c** Analysis of *FLT3*-ITD^mut^ AR on MNCs from mutated AML patients (*n* = 12), including three cases who remained in CCR at 17 months (range 14–20 months), six who relapsed after a median of 7 months (range 3–8 months) from diagnosis and three who death in induction therapy. AR allelic ratio, MNCs mononuclear cells, CCR continuous complete remission.

We studied the subclonal architecture of *FLT3*-ITD^mut^ AML, using samples from 38 patients with a *FLT3*-ITD^mut^ AML. The median AR for *FLT3*-ITD^mut^ in BM-MNCs was 0.34 (range 0.03–1.11) (data not shown). For the purpose of this study, we included three cases with FLT3-ITD^mut^ AR below the <0.05 threshold, which we classified as *FLT3*-ITD^mut^ after confirmation of the ITD size by cDNA analysis. *NPM1* mutations were identified by capillary electrophoresis in 19 AML patients (50%) (median AR:0.75, range 0.44–0.93) (Fig. 1a). The comparison of ARs in 19 *FLT3*-ITD^mut^/*NPM1* double-mutant AML, showed that *FLT3*-ITD^mut^ allelic burden was heterogeneous and significantly lower than that of *NPM1* (median *FLT3*-ITD^mut^ AR: 0.36 vs *NPM1* AR: 0.75, p = 0.001, Fig. 1a), with no correlations between the mutation loads of the two genes in individual patients (Spearman *r* = 0.07, *p* = 0.74). The ITD size was highly variable, with a median of 42 bp (range 16–186 bp). These data confirm that *FLT3*-ITD^mut^ is present at heterogeneous levels at AML diagnosis, different from *NPM1* mutations that are typically heterozygous^18^.

Looking for differences predictive of outcome, we then analysed the mutation profiles of 12 of these patients at diagnosis, using the Oncomine™ Myeloid-Research Assay panel. Clinical and molecular characteristics of these patients are shown in Table 1. We identified a median of three additional mutations per *FLT3*-ITD^mut^ AML (range, 1–7), with *N-RAS* (5 of 12, 41%), *RUNX1* (4 of 12, 33%) and *DNMT3A* (3 of 12, 25%) as the most frequent alterations. Concomitant mutations (number or type) were not predictive for the presence of a *FLT3*-ITD^mut^ subclone (*n* = 3, AR < 0.05) or of outcome (Fig. 1b). Also, the number of concomitant mutations did not impact on the probability of relapse (median cumulative incidence of relapse (CIR) on 4.5 months in patients with ≤2 mutations vs 5 months in patients with ≥3 mutations, *p* = 0.794).

BM-MNC follow-up samples were available for nine patients, including three patients who remained in continuous complete remission (CCR) at 17 months (range 14–20 months), and 6 who relapsed after a median of 7 months (range 3–8 months) from diagnosis. As shown in Fig. 1c, *FLT3*-ITD^mut^ were not detectable by capillary electrophoresis in CR samples, both in patients who remained in CCR and in those who later relapsed, where the FLT3-ITD^mut^ burden increased in five of six cases. On patient lost the FLT3-ITD^mut^ clone at relapse.

### Immunophenotype profiles and FLT3-ITD allele burden in highly purified LPCs at AML diagnosis

We performed additional molecular and immunophenotypic studies in samples sorted from the 12 *FLT3*-ITD^mut^ patients.

**Fig. 2:**
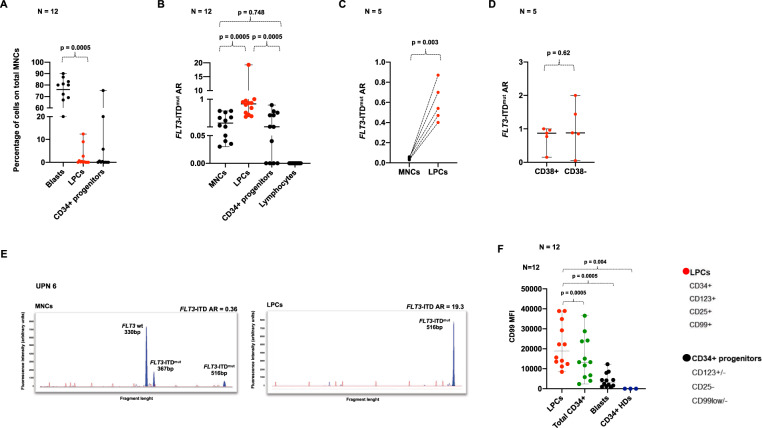
FLT3-ITD^mut^ AR in primary BM samples analysed at AML diagnosis. **a** Proportion of blasts, LPCs and CD34+ progenitors, defined as the percentage of total MNCs, obtained from AML patients by high-speed sorting at diagnosis (*n* = 12). **b** Analysis of *FLT3*-ITD^mut^ AR in the specific BM subpopulations in AML cases (*n* = 12). **c** Enrichment of *FLT3*-ITD^mut^ load in LPCs purified from AML patients characterized by subclonal *FLT3*-ITD^mut^ mutations, as compared to MNCs (*n* = 5). **d** Analysis of *FLT3*-ITD^mut^ AR in LPCs separated according to CD38 expression in AML patients at diagnosis (*n* = 5). **e** Genescan electropherograms of *FLT3*-ITD^mut^ PCR fragments amplified from MNCs and LPCs DNA from UPN 6 at AML diagnosis. We observed two different *FLT3*-ITD^mut^ clones (duplication of 37 and 186 bp) in BM-MNCs cells, while LPCs displayed only the large *FLT3*-ITD^mut^ clone in a homozygous state. The PCR amplicon size of *FLT3* wild-type allele corresponds to 330 bp. **f** Mean fluorescence intensity (MFI) of CD99 antigen on LPCs, total CD34+ cells and primary blasts purified from AML patients by high-speed sorting at diagnosis (*n* = 12). CD99 MFI was also evaluated on CD34+ cells obtained from BM samples of 3 HDs. The antibody combination used for cell sorting is indicated. P values were calculated by a paired Student’s *t*-test. LPCs leukemic precursor cells, AR allelic ratio, MNCs mononuclear cells, HDs healthy donors.

The number of CD34/CD123/CD25/CD99+ LPCs, which corresponds to the LAIP of *FLT3*-ITD^mut^ AML, was assessed using cytofluorimetry, and accounted for 0.15% of BM-MNCs only (median, range 0.001–12.4%), while CD34+ progenitors represented 0.07% of BM-MNCs (median, range 0.02–75.2%), *p* = 0.0005 (Fig. 2a). Representative plots of two patients, one with a clear LAIP and one with a minor subclone, are shown in Supplementary Fig. 1.

LPCs were characterized by a significantly higher *FLT3*-ITD^mut^ load, as compared to MNCs (median AR: 0.84, range 0.42–19.3, vs 0.17, range 0.035–0.6 *p* = 0.0005, *n* = 12), and CD34+ progenitors (CD123+/−, CD25-, CD99low/-) (median AR = 0.047, range 0–0.8) (*p* = 0.0005) (Fig. 2b). Lymphocytes sorted according to the CD45high/CD3+/CD14-/CD33-/CD34- immunophenotype, were *FLT3*-ITD negative, as expected. Of note, a small *FLT3*-ITD^mut^ clone, observed in the MNCs of six patients at a median AR of 0.04 (range 0.03–0.06), was highly enriched in the corresponding sorted LPCs (median AR = 0.54, range 0.4–0.87, *p* = 0.003, Fig. 2c). Further sorting of LPCs for CD38 did not enrich for *FLT3*-ITD^mut^ clones (median AR: 0.87, range 0.15–1, vs 0.88, range 0.05–2, *p* = 0.62, Fig. 2d).

Using the LPCs data, we could classify as *FLT3*-ITD^mut^ 3 AML patients, otherwise classified as *FLT3*-ITD wild-type. Two of these patients had a full-blown *FLT3*-ITD^mut^ relapse.

Interestingly, in one patient (UPN 6), characterized by two different *FLT3*-ITD^mut^ clones in the BM-MNCs at diagnosis (duplication of 37 and 186 bp), the LPCs displayed only the larger *FLT3*-ITD clone in a homozygous state, probably deriving from the copy-neutral loss of heterozygosity (CN-LOH_13q_) (Fig. 2e).

Since, as previously shown, CD99 expression is significantly associated with *FLT3*-ITD mutations^19^, we studied CD99 expression levels in the different BM-subpopulations, expressed as mean fluorescence intensity (MFI) (*n* = 9 AML cases). CD99-MFI level was significantly higher in LPCs (median: 15647, range 8419–38958), as compared to CD34+ cells (median CD99-MFI 13305, range 2352–28762) (*p* = 0.005), blasts (median CD99-MFI 4349, range 1605–12283) (*p* = 0.005) and CD34+ cells purified from three healthy donors (HDs) (median CD99-MFI 2.7, range 1.68–3.9) (*p* = 0.004) (Fig. 2f).

In this line, the *FLT3*-ITD^mut^ and *NPM1*^mut^ burden was different in the cell compartments, with *NPM1* mutations homogeneously expressed at high levels both in blasts, LPCs and CD34+ progenitors, and highly variable *FLT3*-ITD^mut^ loads (*p* = 0.006) (Supplementary Fig. 2). This reflects the nature of *NPM1* mutations, which are considered initiating events in AML.

**Fig. 3:**
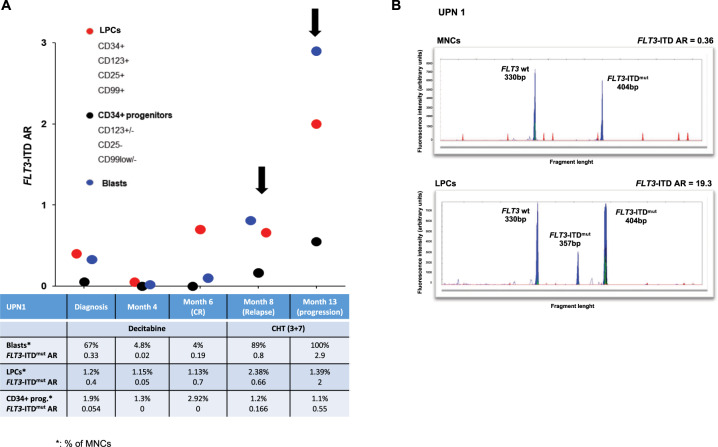
Analysis of *FLT3*-ITD AR in AML during follow-up. **a** Evaluation of the course of bulk leukemic blasts, LPCs-enriched fraction, and CD34+ progenitors subpopulations, purified from AML patient (UPN 1) who achieved CR after 6 cycles of decitabine, relapse and was treated with the “3 + 7” cytarabine and daunorubicin chemotherapy regimen. Although the median proportion of BM-LPCs was 1.2% (range 1.13–2.38%), the *FLT3*-ITD^mut^ load persisted at high levels in these cells at the time of CR, preceding relapse by two months. The relative proportion of the different cell subpopulation and the antibody combinations used for cell sorting are indicated. CR complete remission, LPCs leukemic precursor cells. **b** Genescan electropherograms of *FLT3*-ITD^mut^ PCR fragments amplified from MNCs at first (at 8 months) and second (at 13 months) hematological relapse. One *FLT3*-ITD^mut^ clone (duplication of 74 bp) was present at first relapse, and an additional *FLT3*-ITD^mut^ clone (duplication of 27 bp) emerged at second relapse. *P* values were calculated by a paired Student’s *t*-test. MNCs mononuclear cells.

We were then interested whether the mutation status of sub-populations would be informative of subsequent disease course. We prospectively collected and sorted LPCs and CD34+ progenitors in one of the patients (in UPN1), at diagnosis and at different treatment time-points, after decitabine, and after the following 3 + 7 salvage chemotherapy (Fig. 3). A progressive increase of *FLT3*-ITD^mut^ burden was observed in LPCs as compared to CD34+ progenitors and blast population. In particular, a high *FLT3*-ITD^mut^ allele burden was detectable in enriched LPCs at the time of complete morphologic remission (at 6 months), preceding the first hematologic relapse by 2 months (Fig. 3a). Furthermore, comparing the *FLT3*-ITD^mut^ load in cell subsets, counted as proportion of total MNCs during the disease course, we observed progressive expansion of the *FLT3*-ITD load, leading to AR > 1, probably due to CN-LOH_13q_, while the absolute number of LPCs did not expand (Fig. 3b). These data indicate that *FLT3*-ITD^mut^ clones may persist in LPCs at the time of complete remission, and represent the treatment-resistant *FLT3*-ITD reservoir, later driving disease relapse. In particular, at the time of the third relapse, an additional *FLT3*-ITD^mut^ clone (ITD of 27 bp) was detected both in blasts and in LPCs compartments, indicating clonal evolution (Fig. 3c).

### Additional mutations in leukemic progenitor cells at diagnosis

**Fig. 4:**
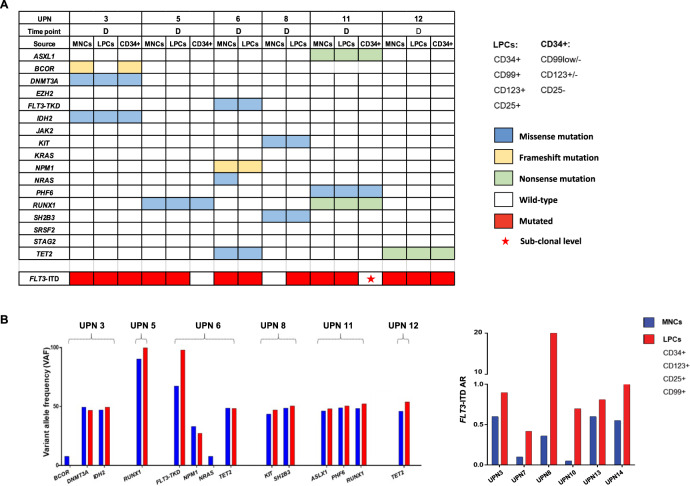
Targeted NGS analyses in AML progenitor cells at diagnosis. **a** Mutational profiling of MNCs and high-speed sorted subpopulations of AML patients at diagnosis (*n* = 6). Patient samples are displayed as columns, D diagnosis, MNCs mononuclear cells. **b** Comparison of variant allele frequencies of mutations detected by NGS and *FLT3*-ITD^mut^ AR studied by capillary electrophoresis, in six diagnostic samples. LPCs leukemic precursor cells, AR allelic ratio, MNCs mononuclear cells. *P* values were calculated by a paired Student’s *t*-test.

To shed light on the molecular heterogeneity of AML blasts, we performed targeted NGS on the cell sub-populations of 6 *FLT3*-ITD^mut^ patients, using the Oncomine™-Myeloid-Research-Assay-panel (Fig. 4). The mutational profile of MNCs was compared to that of LPCs and/or to the CD34+ progenitors. The same mutation pattern was present in both blasts and LPCs in four cases (Fig. 4a). In two cases, with the NGS detection limits of 5%, mutations in *BCOR* and *NRAS* were identified in MNCs only, probably indicating the acquisition of additional events or expansion of the mutated clones during differentiation. Furthermore, by comparing the VAF of mutations by targeted NGS to the *FLT3*^mut^ AR in the different cell populations, we confirmed that while the *FLT3*-ITD burden was again significantly higher in LPCs compared to MNCs, the load of other mutations was similar in the two cell compartments (Fig. 4b).

### Mutation profile in AML relapsing after treatment with tyrosin-kynase inhibitors (TKI) and treatment with CD99

**Fig. 5:**
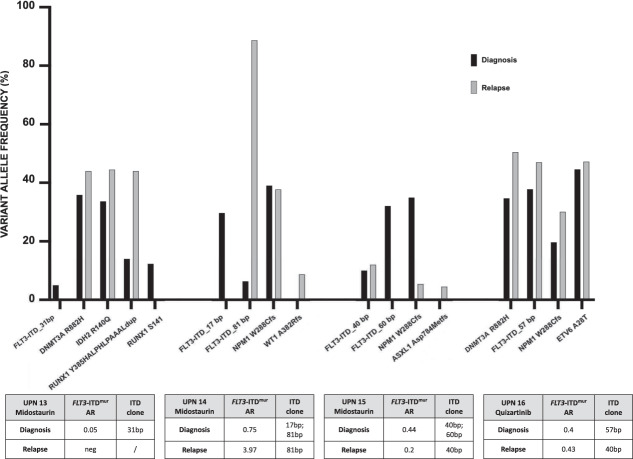
Clinical, biological characteristic, and targeted NGS analyses at diagnosis and relapse of AML patients treated with TKIs (*n* = 4) (Midostaurin in 3 and Quizartinib in 1 pts). TKIs tyrosine kinase inhibitors. AR allele ratio.

TKIs have been recently approved for the treatment of FLT3^mut^ AML. We then studied whether TKI addition to standard chemotherapy (CHT) would modify the mutation profile of AML at relapse. Using the Myeloid Solution panel (SOPHiA GENETICS), we compared the mutation profile at diagnosis and at relapse in 4 *FLT3*-ITD^mut^ samples from patients who relapsed after 3 + 7/midostaurin or quizartinib in combination with CHT, at a median of 10.5 months (range 6–14) from diagnosis (Table 2 and Fig. 5). In two patients characterized by two different *FLT3*-ITD^mut^ clones at diagnosis (UPN14/UPN15) only one clone was retained at relapse after midostaurin, while the third patient became *FLT3*-ITD negative (UPN13). The same *FLT3*-ITD^mut^ pattern was maintained both at diagnosis and at relapse in one patient treated with quizartinib (UPN16). Minor changes in the mutation type and load occurred for other genes (Fig. 5). These data are in agreement with the specific activity of TK, and with the occurrence of FLT3-ITD^mut^ clonal selection at relapse.

Looking at LPCs and CD34 + progenitors, we confirmed enrichment of *FLT3*-ITD mutations in sorted LPCs at diagnosis in UPN 11 (*FLT3*-ITD AR = 0.42), with loss of the mutation at relapse, both in blasts and in CD34/CD123/CD99+ purified cells (supplementary Fig. 3). A second patient (UPN15) was *FLT3*-ITD^mut^ both at diagnosis and at relapse, with expansion of the *FLT3*-ITD^mut^ clone to the homozygous state in LPCs (Supplementary Fig. 3).

**Fig. 6:**
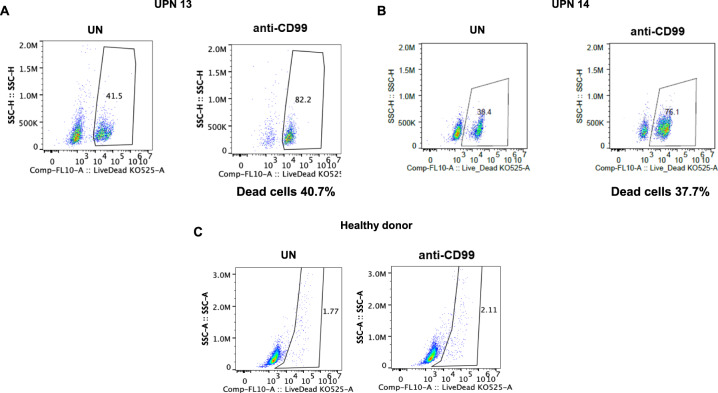
Cytotoxicity of the anti-CD99 mAb on CD34+ cells from two AML patients and one HD. The cytotoxic activity of the anti-CD99 mAb clone 3.2–3 was evaluated on CD34/CD123/CD99+ LSCs sorted from (**a**) UPN 13 and (**b**) UPN 14 at the time of relapse after midostaurin and on BM CD34+/CD99- cells isolated from 1 HD (**c**). The anti CD99 mAb cytotoxic effect was assessed by the Live/Dead assay after 72 h of incubation with the CD99 mAb (25 µg/ml). The proportion of dead cells was calculated by subtracting the value obtained in the absence of treatment (UT) from that of treated cells incubated with the anti-CD99 antibody. HD healthy donor.

Since recent studies demonstrated that CD99 may be a potential therapeutic target in AML^20^, as proof of principle, we tested the activity of the anti-CD99 mAb clone 3.2–3 on LPCs sorted from UPN 13 and UPN 14 at the time of relapse after midostaurin. BM CD34+/CD99- cells isolated from two healthy donors served as controls. As shown in Fig. 6, the anti-CD99 mAb was cytotoxic on enriched LPCs (40.7% and 37.7% dead cells in UPN 13 and UPN 14, respectively, Fig. 6a, b) as compared to untreated cells (UT), while there was no effect on normal CD34+ cells from a healthy donor (Fig. 6c).

## Discussion

We previously showed that the specific CD34/CD123/CD25/CD99 + LPCs phenotype predicts for *FLT3*-ITD^mut^ in AML^9^. Here, we show that the *FLT3*-ITD^mut^ load is significantly enriched in LPCs purified by high-speed cell sorting, as compared to the bulk blast cell population or to CD34+ progenitors isolated at AML diagnosis. Our data confirm that the onset of *FLT3*-ITD^mut^ may be an early event occurring at the stem cell level in AML. This is of clinical relevance, since using the LPCs data we could classify as *FLT3*-ITD^mut^ 3 patients who were under the threshold limits of *FLT3*-positivity in MNC. According to ELN 2017, the *FLT3*-ITD^mut^ AR identifies different prognostic subgroups in AML, and high *FLT3*-ITD^mut^ burden (>0.5) predicts for unfavorable outcome in *NPM1*^mut^ AML^21–24^. The mechanisms behind the variation in AR remains poorly understood, but recent reports suggest that CN-LOH_13q_ may induce high *FLT3*-ITD^mut^ AR^25,26^. Clonal evolution is unfortunately common in *FLT3*-ITD^mut^ AML, with loss of the *FLT3* wild-type allele associated with an aggressive phenotype^27^. In this line, in one of our patients, carrier of two small *FLT3*-ITD^mut^ clones at diagnosis, the LPCs displayed homozygosity for the larger *FLT3*-ITD^mut^ clone at relapse, likely originating from CN-LOH_13q_. Also, *FLT3*-ITD burden was significantly higher in LPCs as compared to blasts and CD34+ progenitors, while the load for other mutations was similar in the different cell populations.

The role of *FLT3*-ITD^mut^ as a minimal residual disease marker is debated, due to the instability at relapse and the technical issues related to the patient-specificity of the ITD sequence^28^. In one of our patients, *FLT3*-ITD^mut^ LPCs were present at the time of complete remission, while BM-MNC were *FLT3*-ITD^mut^ negative, preceding morphological relapse by two months. These data indicate that LPCs may represent the leukemic reservoir driving disease relapse. Our data highlight the relevance of characterizing the mutational and immunophenotypic profiles of LPCs, also to eventually add targeted drugs. In this setting, TKIs are a successful therapeutic strategy in AML, and in most recent years several *FLT3*-targeted agents have been approved by FDA and EMA^29,30^. The first new molecule approved in nearly two decades has been midostaurin, a multikinase inhibitor active against both *FLT3*-ITD and TKD mutations. Although midostaurin demonstrated high efficacy when combined with conventional chemotherapy in AML, the relapse rate remains high, at about 50% at a median of two years of follow-up^5^. In this setting, the results from our study demonstrate that TKIs treatment may impact on clonal evolution of *FLT3*-ITD^mut^ AML, downregulating specific clones. These data, underscore the importance of repeated mutation testing for *FLT3*-ITD^mut^ to distinguish patients where TKI may induce long-lasting remission from those where relapse may originate from subclones, which may be *FLT3*-wild type.

One of the mechanisms of treatment failure may be the persistence of *FLT3*-ITD^mut^ LPC, as a potential source of relapse. These cells are characterized by overexpression of CD99, as shown by significantly increased MFI compared to CD34+ progenitor cells. To overcome midostaurin resistance and try to target disease-initiating leukemic stem cells, as proof of principle, we tested a CD99-specific antibody on primary patient samples collected at the time of relapse following midostaurin. This treatment was cytotoxic on CD34/CD99+*FLT3*-ITD^mut^-LPCs, resistant to midostaurin, while there was no effect on healthy CD34+ cells. One of our patients became *FLT3*-ITD^mut^ negative at relapse. Loss of *FLT3*-ITD^mut^ at disease recurrence has been reported in 25% of patients, and this figure may increase after treatment with TK inhibitors. The CD99 antibody may represent an attractive treatment option in these cases^21^.

In conclusion, we showed that *FLT3*-ITD^mut^ are present at LPC level and may represent a primary event in leukemogenesis. According to these data, we suggest that the identification of *FLT3*-ITD^mut^ LPCs driving therapy resistance and/or disease progression in AML may allow to design novel disease-eradicating therapies and eventually improve disease outcome.

## Supplementary information

Supplementary Figure 1, Supplementary Figure 2, Supplementary Figure 3

Supplementary Figure 1, Supplementary Figure 2, Supplementary Figure 3

Reproducibility checklist
