# Supplementary material for: Characterization of FLT3-ITDmut acute myeloid leukemia: molecular profiling of leukemic precursor cells
Source: Blood Cancer J. 2020 Aug 25;10(8):85. doi: 10.1038/s41408-020-00352-9 (PMC7447750; doi:10.1038/s41408-020-00352-9)
Supplement: Supplementary file 1 — Supplementary Figure 1, Supplementary Figure 2, Supplementary Figure 3 [file 41408_2020_352_MOESM1_ESM.pptx]

## Slide 1
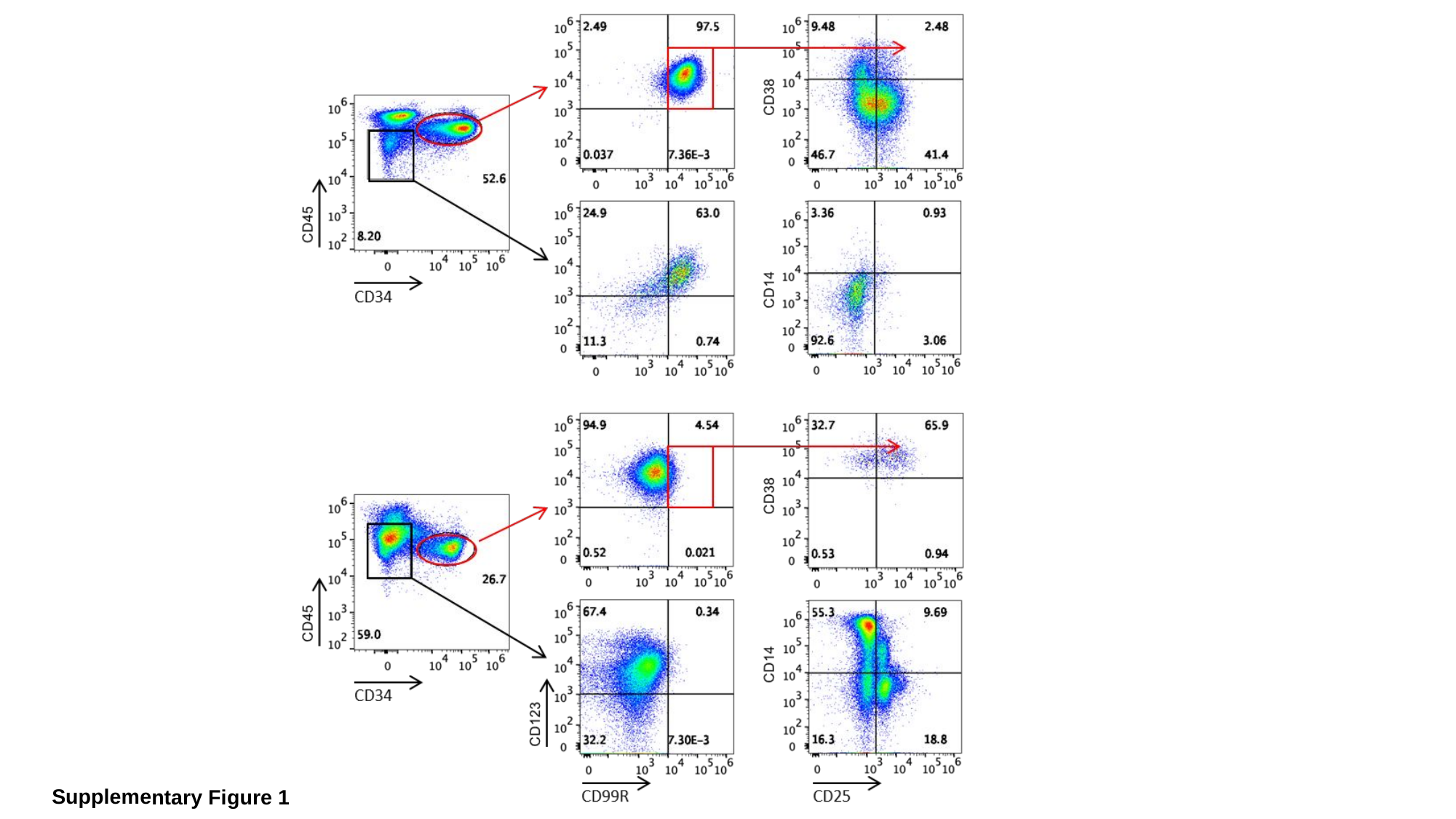

Supplementary Figure 1

## Slide 2
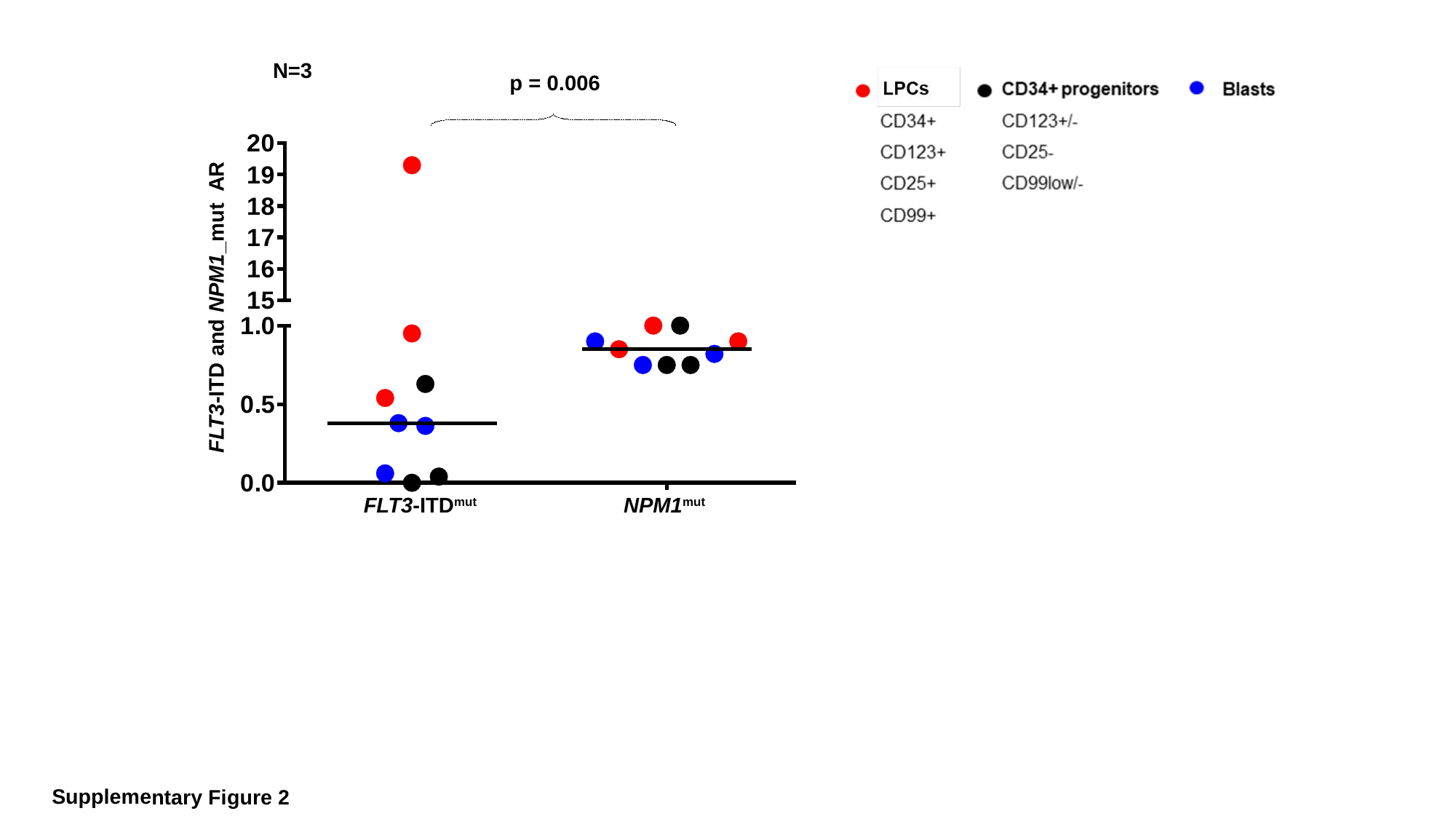

N=3
p = 0.006
FLT3-ITD and NPM1_mut AR
| FLT3-ITDmut |
| --- |
| NPM1mut |
| --- |
Supplementary Figure 2

## Slide 3
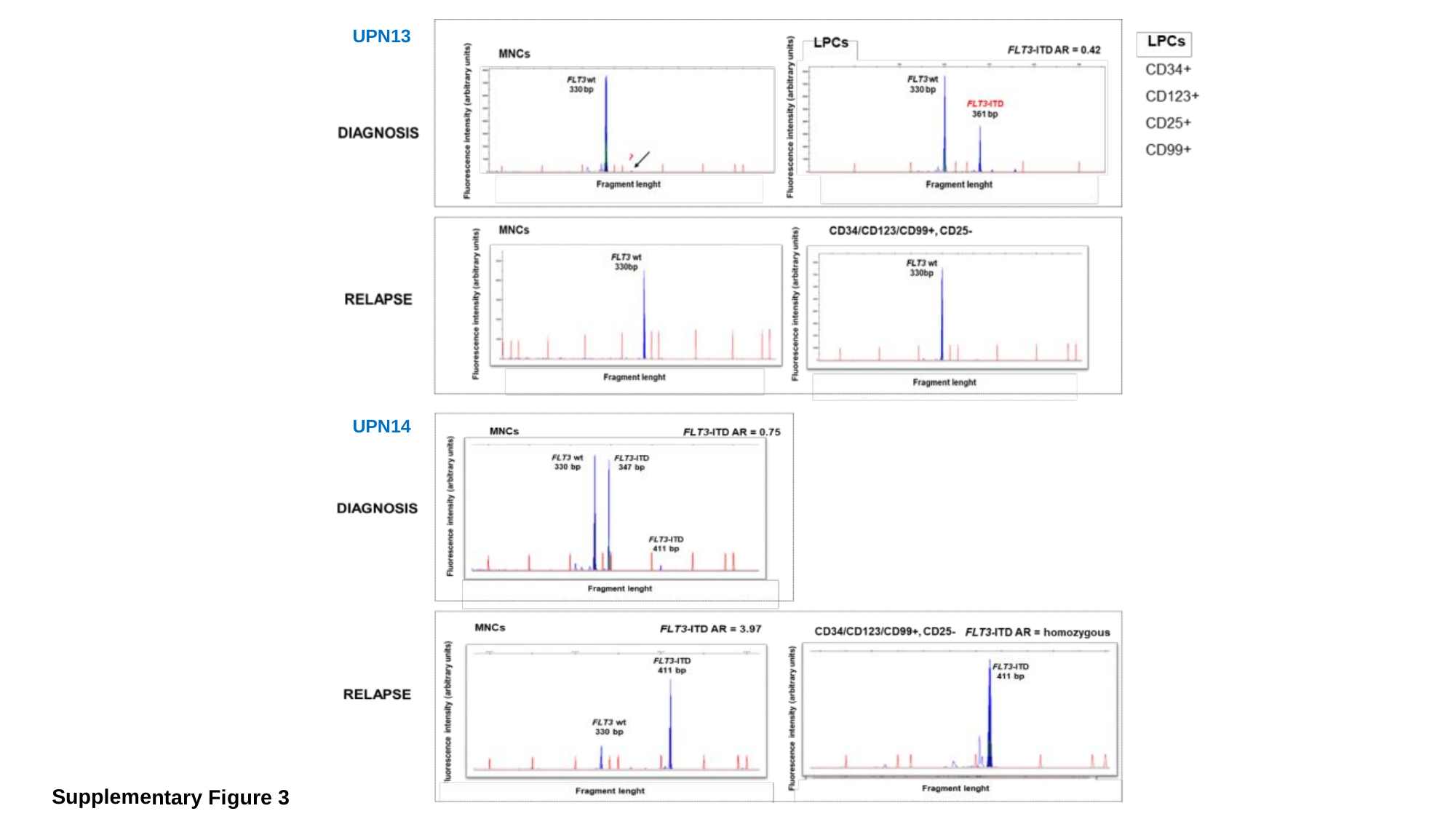

UPN13
UPN14
Supplementary Figure 3
