# Supplementary material for: Characterization of FLT3-ITDmut acute myeloid leukemia: molecular profiling of leukemic precursor cells
Source: Blood Cancer J. 2020 Aug 25;10(8):85. doi: 10.1038/s41408-020-00352-9 (PMC7447750; doi:10.1038/s41408-020-00352-9)
Supplement: Supplementary file 2 — Supplementary Figure 1, Supplementary Figure 2, Supplementary Figure 3 [file 41408_2020_352_MOESM2_ESM.docx]

**Supplementary figures legend**

**Supplementary figure 1** **Immunophenotyping by MFPC analysis of 2 representative AML samples at diagnosis** that shown the expression of CD123 vs CD99R and CD38 vs CD25 within the CD34+ population. Co-expression of CD123/CD25/CD99+ antigens within the CD34+ population are highlighted by the red quadrangle. MFPC: multiparameter flow cytometry.

**Supplementary figure 2** ***FLT3*-ITD^mut^ and *NPM1*^mut^ AR analysis in BM-MNCs samples of AML patients at diagnosis.** *FLT3*-ITD_mut_ and *NPM1*^mut^ AR was evaluated in the different cell compartments in 3 AML cases.

The *FLT3*-ITD^mut^ and *NPM1*^mut^ AR were defined as the ratio of the area under the curve of *FLT3*-ITD^mut^ and *FLT3* wild type alleles. The antibody combination used for cell sorting is indicated. The cell populations analyzed are indicated by different colors (i.e.: red for LPCs, black for CD34+ progenitors, blue for blasts).

P values were calculated by a paired Student's t-test. AR: allelic ratio, MNCs: mononuclear cells.

**Supplementary figure 3** **Electropherograms of PCR fragments obtained from *FLT3*-ITD^mut^ analysis** on MNCs and CD34/CD123/CD99+ LPCs purified from UPN 13 and UPN 14 AML patients at diagnosis and relapse. MNCs: mononuclear cells, LPCs: leukemic precursors cells.
